# What every psychiatrist should know about PANDAS: a review

**DOI:** 10.1186/1745-0179-4-13

**Published:** 2008-05-21

**Authors:** Germana Moretti, Massimo Pasquini, Gabriele Mandarelli, Lorenzo Tarsitani, Massimo Biondi

**Affiliations:** 1 Department of Psychiatric Sciences and Psychological Medicine, "Sapienza" University of Rome, Viale dell'Univeristà 30, 00185, Rome, Italy

## Abstract

The term Pediatric Autoimmune Neuropsychiatric Disorders Associated with Streptococcus infections (PANDAS) was coined by Swedo et al. in 1998 to describe a subset of childhood obsessive-compulsive disorders (OCD) and tic disorders triggered by group-A beta-hemolytic Streptococcus pyogenes infection. Like adult OCD, PANDAS is associated with basal ganglia dysfunction. Other putative pathogenetic mechanisms of PANDAS include molecular mimicry and autoimmune-mediated altered neuronal signaling, involving calcium-calmodulin dependent protein (CaM) kinase II activity. Nonetheless the contrasting results from numerous studies provide no consensus on whether PANDAS should be considered as a specific nosological entity or simply a useful research framework. Herein we discuss available data that could provide insight into pathophysiology of adult OCD, or might explain cases of treatment-resistance. We also review the latest research findings on diagnostic and treatment.

## Introduction

Several studies provide compelling evidence of cortico-subcortical involvement in the pathogenesis of obsessive-compulsive disorder (OCD) [1]. Data emerging from morphological and functional neuroimaging studies suggest specific alterations at the level of orbitofrontal-caudate-thalamic circuits [2,3]. Patients with adult-onset OCD often have a history of ischemic stroke or traumatic brain injury involving the basal ganglia [4-6]. Moreover, indirect evidence of basal ganglia involvement in OCD comes from observations that the symptoms of OCD regress after surgery for cingulotomy and capsulotomy, interventions that disconnect the frontal cortex from basal ganglia [7,8].

Despite advances in the knowledge of the pathogenesis of OCD, little is known about the causative mechanisms underlying specific alterations. Observations of patients with rheumatic fever who had Sydenham's chorea manifesting with classic OCD symptoms have suggested a possible etiological link between group A β-hemolytic streptococcus (GABHS) infection in a subset of OCD patients [9-11]. GABHS has also been implicated in the development of Tourette syndrome [12-14] and autism in children [15].

These clinical reports engendered considerable interest in a possible streptococcal-triggered etiology for OCD. In 1998 the National Institute of Mental Health instituted a research group that sought to characterize a subgroup of children with OCD and tic disorders (TD), namely "pediatric autoimmune neuropsychiatric disorders associated with streptococcal infections" (PANDAS) [16]. These investigators outlined diagnostic criteria, studied several candidate PANDAS patients and proposed a possible pathophysiologic mechanism according to which a susceptible host might produce antibodies against GABHS that cross-react with neuronal tissue [16]. This mechanism resembles what happens in GABHS post-infection sequelae glomerulonephritis and rheumatic fever. Evidence suggesting a possible association between OCD and GABHS infection also in adults, comes from three case reports describing the abrupt onset of OCD due to GABHS infection [17-19]. In all cases the infection and obsessive-compulsive symptoms both promptly responded to antibiotics.

Recent evidence suggests that specific antibodies targeted to the dominant epitope of GABHS (N-acetyl-beta-D-Glucosamine) might influence neuronal signal transduction thus causing alterations in behavior and movement control. Accordingly, sera from some patients with Sydenham's chorea [20] or PANDAS [21] contain antibodies that induce calcium-calmodulin dependent protein (CaM) kinase II activity.

Despite growing support for an association between GABHS and OCD, the causal relationship between GABHS infection and OCD, its pathophysiology, and its possible clinical implication remain highly controversial. In this paper we will review human studies aimed at verifying the PANDAS construct. Our review focuses primarily on the pathogenetic mechanisms underlying the development of PANDAS. An important unanswered question is whether some patients currently treated for OCD are actually undiagnosed PANDAS in childhood. Knowing more about the pathogenesis of PANDAS might improve our insight into pathogenetic mechanisms of treatment-resistant OCD.

### PANDAS: historical perspective

In 1994 Susan E. Swedo reported the case of a 9-year-old girl with rheumatic chorea and OCD whose neuropsychiatric symptoms responded to plasma exchange [9]. In the ensuing years a growing interest focused on the neuropsychiatric features of rheumatic fever and reports described patients with Sydenham's chorea who in up to 70% of cases manifested obsessive-compulsive symptoms, apparently indistinguishable from those of classic OCD [9,10]. The relationship between GABHS and Sydenham's chorea has long been documented in the medical literature [22]: streptococcal peptides stimulate specific lymphocyte immune responses producing antibodies that might in turn cross-react with various host epitopes, through the mechanism known as molecular mimicry [23,24]. In analogy to Sydenham's chorea, infections with GABHS may trigger autoimmune responses that cause or exacerbate some cases of child-onset OCD, TD or Tourette syndrome [25].

The first systematic attempt to identify and define a nosological entity characterized by pediatric OCD or TD, triggered by an infection and with a supposed autoimmune pathogenesis, dates back to 1995. In this year Allen and co-workers described 4 patients in whom OCD or Tourette syndrome manifested or worsened after GABHS infection (two cases) and viral infection (two cases), and responded to treatment with plasmapheresis, intravenous immunoglobulin (IVIG) or immunosuppressive doses of prednisone [25]. To summarize the essential features of this subgroup of patients with OCD or TD, Allen et al. coined the acronym PITANDs (pediatric infection-triggered, autoimmune, neuropsychiatric disorders). As possible triggers of the neuropsychiatric manifestations they originally included along with GABHS infection, viral or other bacterial infections [25].

In a later study in 1998, Swedo et al. reappraised and extended the diagnostic criteria for PITANDS, no longer mentioned viral or other bacterial infections and hypothesized the existence of PANDAS [16]. They proposed five diagnostic criteria 1) the presence of OCD or a tic disorder or both, 2) pediatric onset, 3) episodic course of symptom severity with abrupt onset or dramatic symptom exacerbations, 4) temporal association with GABHS infection 5) and association with neurological abnormalities during symptom exacerbations [16]. Whereas the PITANDs hypothesis focused generally on a possible association between "an antecedent or concomitant infection" and neuropsychiatric manifestations, the diagnostic criteria for PANDAS were restricted to GABHS infection.

In a systematic clinical evaluation of 50 children who met the diagnostic criteria for PANDAS, Swedo and colleagues found that these patients typically had a young age at illness onset, an abrupt onset of neuropsychiatric symptoms and frequently manifested neuropsychiatric comorbidities (attention deficit hyperactivity disorder 40%, major depressive disorder 36%, overanxious disorder 28%, separation anxiety disorder 20%, enuresis 12% [16]. GABHS infection preceded 45 (31%) of 144 exacerbations of TD or OCD. Moreover, the onset of behavioral symptoms (irritability, emotional lability, tactile/sensory defensiveness, motor hyperactivity, deterioration in handwriting) was typically associated with exacerbation of OCD or tics, triggered by GABHS infections [16], also in patients who had Sydenham's chorea [9].

Subsequent studies investigating the PANDAS hypothesis yielded controversial results. Some seemed to confirm the association between GABHS infection and OCD or TD exacerbations [26,27], whereas others failed [28,29]. Circumstantial evidence indicating PANDAS as an autoimmune disorder came also from the presence of anti-neuronal (anti-brain, anti-basal ganglia) antibodies in children with PANDAS [30-32] or children with Tourette syndrome [33-35]. Again other studies failed to identify significant differences into auto-antibody levels between patients with PANDAS and controls [36-38]. The discrepancies among the various researches presumably arise partly from methodological problems: for example, the use of rabbit neural antigens having low homology with the human isoforms instead of human antigens [38]. Strong support for PANDAS as an immune-mediated disorder comes from the excellent response of children with PANDAS to immunotherapies (plasma exchange and IVIG) [39].

Prompted by a report that antibiotic prophylaxis diminished recurrences of rheumatic fever, some investigated a possible analogous outcome in patients with PANDAS. The first trial with oral penicillin was unsuccessful [40]. Another prospective longitudinal trial of azithromycin or oral penicillin in 23 children with PANDAS showed that antibiotic prophylaxis, with both molecules, effectively decreased streptococcal infections and neuropsychiatric symptoms exacerbations among children with PANDAS [41].

### Diagnostic issues

As well as stimulating considerable attention the PANDAS hypothesis has generated controversy and skepticism. A major criticism is although the currently proposed diagnostic criteria focus on the occurrence, onset, severity and course of tic or obsessive compulsive symptoms they may fail to distinguish PANDAS from other phenotypes of OCD or tics, and to some degree even from Sydenham's chorea [42,43]. A childhood onset of symptoms (second criterion) may lack the specificity needed to distinguish PANDAS from Tourette syndrome, because in up to 75% of TD cases the manifestations begin during the prepubertal period [44]. Moreover, in a series of 80 patients with TD, 53% of the sample reported a sudden onset of illness [45]. Furthermore, the specificity of a sudden and dramatic onset (third criterion) for the PANDAS subgroup of OCD or TD or both has been questioned because some reports describe cases of sudden-onset of adult OCD or TD after GABHS [17,19,46,47]. Most children enrolled in PANDAS studies manifested several neuropsychiatric comorbidities especially attention deficit hyperactivity disorder, anorexia nervosa, dystonia, trichotillomania, major depressive disorder, or separation anxiety disorder [16,30,31,41]. Whether these manifestations are independent, secondary to the development of PANDAS or, at least in some cases, could share a common pathogenetic pathway is unclear. Obviously, the presence of neuropsychiatric comorbidities limits the discriminating specificity of the diagnostic criteria, but in childhood neuropsychiatric disorders this is the rule rather than the exception. The presence of neurological abnormalities (fourth criterion) has been often referred to as the presence of choreiform movements (reported in up to 95% of patients with PANDAS in the acute phase), hence becoming a specific criterion [16]. Those supporting the PANDAS hypothesis have been excluding choreic movements as possible neurological manifestation to avoid possible diagnostic overlap between PANDAS and Sydenham's chorea, who often present OCD or TD comorbidities [10,48]. Some authors suggested that the PANDAS subgroup could represent an attenuated form of Sydenham's chorea and that a dysfunction in the basal ganglia could be a common pathogenetic pathway between choreiform movements and overt chorea [42,49]. Subsequent studies nevertheless showed that choreiform movements (elicited exclusively by a clinician on a neurological examination disclosing stressed posture) could be reliably distinguished from choreatic movements (rapid, involuntary, continuously increasing arrhythmic movements that are present continuously and increase during unrelated voluntary movements) [49,50]. Finally the temporal association (fifth criterion) between GABHS infection, whose incidence in school-age children is high, and the onset or the exacerbation of neuropsychiatric symptoms does not necessarily mean causality, the question awaits an answer from further controlled prospective studies. Streptococci were initially associated with Kawasaki disease and Henoch-Schönlein purpura, but controlled studies eliminated bacteria as a causal factor [42]. Streptococci are not the only infectious agents implicated in Tourette syndrome, other pathogens putatively involved include Borrelia burgdorferi and Mycoplasma pneumoniae [51].

A recent study has shown that antibody test reactions for Mycoplasma pneumoniae differ significantly in patients with Tourette syndrome and healthy controls (59% vs. 3%) [51]. Even though most reports involve GABHS, these data suggest that the autoimmune process underlying post-infective autoimmune neuropsychiatric symptoms may be triggered not only by streptococci but also by other infectious agents.

### Physiopathology of GABHS infections

GAHBS is a Gram-positive, extracellular bacterium of spherical to ovoid shape, and is one of the most frequent human pathogens. Several clinical manifestations have been associated with acute GABHS infections, including pharyngitis (strep throat), scarlet fever, impetigo and cellulitis [24]. GABHS produces several extracellular virulence factors including streptolysin S and O, hyaluronidase, opacity factor, NADase and M-like proteins. M protein is the major surface protein and occurs in more than 100 antigenically distinct types, being the basis for the serotyping of strains with specific antisera [24]. Bacterial cell wall M proteins have been found to mimic several cardiac proteins and the group-specific carbohydrate of GABHS resembles the glycoprotein of heart valves. Indirect evidence suggests that M6 and M19 proteins may share common epitopes with brain structures [52]. Group A streptococci also elaborate, to varying degrees, a polysaccharide capsule composed of hyaluronic acid. The description of new virulence factors, not present in the earlier strains, together with a significant increase in the incidence and severity of infections, has suggested that GABHS genome has re-arranged over time. Evidence in recent years suggests that new phage-encoded virulence factors will be identified by sequencing the genomes of additional GABHS strains [53].

An individual's vulnerability to infection-triggered autoimmune disorders depends crucially on genetics. Family-based studies support a genetic predisposition to rheumatic fever [54]. Rheumatic fever is an inflammatory disease that can involve heart, joints, skin and brain. Sydenham's chorea is the most frequent neurological manifestation of rheumatic fever and is characterized by rapid, uncoordinated jerking movements affecting primarily face, feet and hands. Rheumatic fever is believed to be caused by antibody cross-reactivity. This cross-reactivity is a Type II hypersensitivity reaction often referred to as molecular mimicry [54]. Substantial evidence argues for molecular mimicry also in Sydenham's chorea, and anti-GABHS antibodies could cross-react with neuronal tissue [54].

A pioneering study on children with Sydenham's chorea found that 46.6% of sera from 30 patients reacted with neuronal cytoplasm of human caudate and subthalamic nuclei and the presence of anti-neuronal antibody was associated with the severity and duration of clinical attacks [55]. Several subsequent studies investigated the presence of anti-neural or anti-brain antibodies in movement disorders. Antineural antibodies directed against caudate nuclei were found in 10 of 11 patients with Sydenham's chorea [56]. In a later study, Church and colleagues found higher titers of anti-basal ganglia antibodies in patients with acute Sydenham's chorea than in convalescent patients [57].

### Antineuronal autoantibodies

The presence of systemic anti-basal ganglia autoantibodies has been proposed as possible evidence for an immunological pathogenesis of a subset of OCD. The modulation of intracellular signalling pathways by autoantibodies has been described in myasthenia gravis [58] (autoantibodies to the acetylcholine receptor blocking neuromuscular transmission) and Graves disease [59] (autoantibodies against thyroid-stimulating hormone). Nonetheless basal ganglia, like most CNS structures, are relatively inaccessible to circulating antibodies owing to the presence of the blood-brain barrier (BBB). Although the mechanism by which circulating antibodies or cytokines might gain access to the CNS is unknown, with the exception of the circumventricular/lamina terminalis region, where the BBB is absent [60], a variety of hypothetic mechanisms exist. For example, circulating antibodies could reach the CNS if an inflammation of the meninges causes a local BBB breakdown. Cytokines, probably crossing the BBB at circumventricular organs, can trigger an immune activation on the CNS side of the BBB. Moreover, peripheral B cells that are cross-reactive to a CNS epitope may cause intrathecal production of antibodies [61].

To test the specificity of the association between anti-brain antibodies and the neuropsychiatric symptoms in PANDAS, Pavone and colleagues compared a group of PANDAS children with patients with uncomplicated (without neuropsychiatric manifestations) GABHS active infection [30]. They found a far higher incidence of anti-brain antibodies in serum from children with PANDAS than in those with active GABHS infection (64% vs. 9%) suggesting that the presence of anti-brain antibodies could not be accounted for by GABHS infection alone [30]. Further support for an immune-mediated pathogenesis of OCD in a subset of patients came from a study by Dale et al. that compared anti-basal ganglia antibody (ABGA) titers among patients with OCD and three control groups (neurological patients, uncomplicated GABHS infection, autoimmune disorders) and found significantly higher antibody expression in the OCD group (42% vs. 4%, 2%, and 10% in the three control groups) [31]. In the same study the authors found that the mean CY-BOCS score in the antibody-negative patients was higher than in the antibody-positive patients, and the latter had lower obsessions of hoarding/saving [31]. Others nevertheless also found anti-brain antibodies in healthy controls [14,33]. Two successive studies found no significant differences for ABGA immunoreactivity between patients with PANDAS vs. controls [36] and between children who met PANDAS criteria and two control groups (healthy controls and patients with TD) [37]. These discrepancies in autoantibody findings could reflect the methods used for antibody detection: enzyme-linked immunosorbent assay (ELISA) and western blotting which can alter the conformation of the antigens and could therefore affect antibody-antigen interactions [31].

Despite existing evidence of brain-specific antibody reactivity, and the isolation of antibodies against basal ganglia evoked by streptococcal cell wall, the mechanism by which molecular mimicry results in a behavioral or movement alteration is still unclear. Recent work by Kirvan and colleagues suggests that the pathogenesis of PANDAS and Sydenham's chorea might involve immune-mediated altered neuronal signaling [20,21]. These investigators first demonstrated that monoclonal antibodies derived from patients with acute Sydenham's chorea and targeted to N-acetyl-beta-D-glucosamine (GlcNAc), the dominant epitope of GABHS, reacted with human lysoganglioside GM1. This lysoganglioside influences neuronal signal transduction [62]. Moreover the autoantibody 24.3.1, from sera of patients with acute Sydenham's chorea induced CaM kinase II activity, whereas serum obtained from convalescent patients did not [20]. A recent work from the same group reported that antibodies which react with lysoganglioside GM1 and induce CaM kinase II activation in neuronal cells are present in PANDAS [21]. Using competitive-inhibition ELISA Kirvan et al. found that soluble lysoganglioside GM1 inhibited 73% of sera from patients with PANDAS binding to GlcNAc (conjugated to bovine serum albumin) but only 23% of sera from controls (OCD, tic disorders, attention deficit hyperactivity disorder, patients not meeting PANDAS criteria) [21]. Moreover, using human neuroblastoma cell cultures, they showed that PANDAS sera specifically induced antibody-mediated activation of CaM kinase II (75% percent of acute PANDAS sera), the degree of activation being superior to non-PANDAS sera and inferior to chorea sera. PANDAS sera depleted of IgG did not activate CaM kinase II. Notably the degree of activation of CaM kinase II was highest in PANDAS patients with isolated tics.

Current data emerging for patients with chorea, PANDAS and OCD seem to suggest that CaM kinase II could be an intracellular mediator of behavioral and motor manifestations in some neuropsychiatric disorders. Along a continuum of activation from low levels (e.g. non-PANDAS OCD) to extremely high levels (rheumatic chorea), CaM kinase II activity seems to be associated in non-PANDAS OCD with simple neuropsychiatric manifestations and in rheumatic chorea with frank motor alterations. No studies have yet shown whether the physiological systems activating this signal cascade interact with possible disease-related (autoimmune ?) triggers. If they do, these interactions could be a new target for possible pharmacological approaches in disorders such as OCD and choreiform disorders.

### Peripheral markers

The research for a possible susceptibility marker for PANDAS mostly focused on identifying peripheral markers. Among proposed peripheral markers of PANDAS susceptibility is monoclonal antibody directed against a non-HLA B-cell marker known as D8/17. This antibody is an IgM first isolated from fusions of spleen cells from mice that had been repeatedly immunized with human B-cells from patients with confirmed rheumatic fever [63,64].

In a study investigating D8/17 in PANDAS, Swedo and colleagues compared 27 children who met the diagnostic criteria with 9 patients with Sydenham's chorea and 24 healthy controls, and found a significantly higher percentage of B cells that bind D8/17 monoclonal antibody in children with both diseases than in controls (89% in Sydenham's chorea, 85% in PANDAS, 17% in controls) [65]. Another study of patients with child-onset OCD or Tourette disorder found 100% positive reactions for D8/17 in patients compared with 5% in the control group [13]. Subsequent studies investigated D8/17 positive B-cells in obsessive-compulsive spectrum disorders, as well as in other neuropsychiatric disorders. High percentages of B-cells expressing D8/17 were found in patients with autism (78%) [15], anorexia nervosa (100%–81%) [66,67], adult OCD (59%–92%) [68,69], tics (61%) [70] and trichotillomania (59%) [68]. Recent studies that used more accurate methods (flow cytometry) nevertheless failed to replicate these results [71,72]. This discrepancy may be due, at least in part, to the difference in the methods used in these studies, but also to the molecular characteristics of the antibody. The antibody that binds to D8/17 is an IgM, known to be relatively unstable and difficult to purify.

Preliminary evidence suggests that D8/17 antigen immunoreactivity may reflect different psychopathological characteristics among patients with obsessive-compulsive spectrum. In a study on repetitive behaviors in autism Hollander and colleagues investigated the presence of D8/17 antigen in a sample of 18 children with autism. They found that the D8/17-positive patients had significantly higher mean children Yale-Brown obsessive compulsive scale (CY-BOCS) compulsion scores than the D8/17-negative patients [15]. These results suggest that psychopathological characteristics could differ in the various clinical subgroups of patients with OCD according to the underlying pathogenetic mechanisms.

### Neuroimaging

In recent years, evidence arising from morphological and functional neuroimaging studies have linked OCD with dysfunction in frontal-subcortical circuits. Strong evidence exists of orbitofrontal cortex involvement but other areas implicated in the pathogenesis of OCD include the anterior cingulate gyrus, amygdala, insula, thalamus, striatum, lateral frontal and temporal cortices [1-3]. Several studies with positron emission tomography (PET) reported increased glucose metabolism in the orbitofrontal cortex, caudate, thalamus, prefrontal cortex and anterior cingulate among patients with OCD [73-75]. Current knowledge on the pathophysiology of OCD, despite suggesting an involvement of discrete brain regions, is far from concluding that these abnormalities are the cause of OCD or just an epiphenomenon [2].

In 1996 Giedd et al. first described an association between abrupt exacerbation of OCD symptoms after GABHS infection and an enlargement of basal ganglia [76]. Relatively few imaging studies have investigated CNS alterations in SC, most studies found no pathological changes on MRI. An MRI study of 24 children with SC, however, found volumetric abnormalities in caudate, putamen and globus pallidus [77]. Another study in a patient with Sydenham's chorea detected striatal abnormalities (increased signal intensity on T2-weighted images involving the putamen, globus pallidus, and the head of the caudate nucleus bilaterally) that reversed after recovery [78]. A subsequent longitudinal study with MRI compared 34 patients who met PANDAS criteria with 82 healthy controls and found a significant enlargement of caudate, putamen and globus pallidus in the patients [79]. Interestingly, immunomodulatory treatment (plasma exchange and IVIG) normalized this difference, suggesting that basal ganglia abnormalities are reversible. The same study found no correlation between symptom severity and basal ganglia volume [79].

Further longitudinal studies monitoring the CNS changes such as autoimmune vasculitis and edema and OCD symptoms that are supposed to follow GABHS infection are needed to assess a possible causal role and the involvement of specific CNS regions [80].

### Therapeutic strategies

The neurobiological mechanisms underlying the pathophysiology of OCD remain an intense area of research. One of the most accepted theories supports an alteration of serotonergic brain pathways, mainly because serotonin reuptake inhibitors achieve better clinical efficacy than other pharmacotherapeutic agents [81]. Double-blind, placebo-controlled trials have shown the efficacy of clomipramine and selective serotonin reuptake inhibitors (SSRI) in the treatment of adult OCD [81]. Although fewer, but consistent observations, suggest that clomipramine and SSRI may be equally effective in the treatment of childhood OCD, only clomipramine, fluvoxamine and sertraline have been approved by the FDA for child and adolescent OCD.

Several lines of evidence indicate that an optimal treatment for OCD is combined pharmacotherapy and cognitive behavioral therapy (CBT) [82]. Despite the advances in pharmacological and psychotherapeutic approaches, up to 40–60% of treated patients are still non-responders or their response is unsatisfactory [83].

Some reports suggest that OCD or tics manifesting in patients with PANDAS respond to serotonergic drugs and combined therapy [84]. CBT and serotonergic drugs have proven efficacy, whether or not the symptomatology is triggered by a GABHS infection. Even in the PANDAS subgroup many patients have an unsatisfactory response. The true percentage of non-responders remains difficult to define but probably approaches that in the non-PANDAS subgroup.

When standard treatments fail and symptoms are severe and disabling, Swedo and colleagues proposed immunomodulatory interventions, tailored to the presumed pathophysiology [84]. In a longitudinal double-blind placebo-controlled trial of 29 children with PANDAS, plasma exchange, IVIG or sham IVIG proved better than placebo in reducing OCD symptoms at 1-month follow-up (58% improvement with plasma exchange, 45% with IVIG) and tics (49% improvement with plasma exchange, 19% with IVIG) as measured by CY-BOCS and Tourette syndrome unified rating scale [39]. The improvements remained stable at 1 year follow-up, and were all statistically significant (p < 0.05) with the exception of tics in the group treated with IVIG. Whereas standard therapies (SSRI, cognitive behavioral therapy) have proved efficacious in the PANDAS subset of OCD and TD, immunotherapies were ineffective in children with resistant OCD without a history of GABHS infection [85], suggesting that immunotherapy is specific for PANDAS thus supporting the presumed pathophysiology.

In a prospective longitudinal study 12 children who met the diagnostic criteria for PANDAS, were treated with penicillin or amoxicillin (5 patients), amoxicillin and clavulanate (1 patient), or cephalosporin (6 patients) during acute exacerbation of neuropsychiatric symptoms. In all patients antibiotic therapy effectively resolved OCD, the anxiety symptoms and tics within on average 14 days [26]. Penicillin prophylaxis has proven effective in preventing recurrences of rheumatic fever, and the American Heart Association recommend the use of oral penicillin 250 mg twice a day for prevention [86]. Because of the hypothesized pathophysiologic similarities between Sydenham's chorea and PANDAS some have argued that penicillin prophylaxis would also reduce neuropsychiatric exacerbations in children with PANDAS. In the first controlled trial on antibiotic prophylaxis for PANDAS, 37 children who had been previously diagnosed as PANDAS, were randomized to 4 months of penicillin V (twice daily oral 250 mg) followed by 4 months of placebo, or placebo followed by penicillin. In this study oral penicillin failed to provide adequate prophylaxis for GABHS and subsequently for neuropsychiatric symptoms exacerbations [40]. In a subsequent randomized trial Snider and colleagues tried to determine whether the negative results from Garvey were due to inefficacious prophylaxis against GABHS infection, and not to a lack of association between GABHS infection and neuropsychiatric symptoms. The study compared penicillin, considered as an "active placebo", with azithromycin, a drug that had proved efficacious against GABHS infections. In contrast to previous studies, penicillin and azithromycin both effectively decreased GABHS infections and neuropsychiatric exacerbations. The authors therefore concluded that antibiotic prophylaxis may be useful in the management of children with PANDAS [41].

Others later pointed out that the study had several limitations: the small sample size, the use of an "active placebo" and the retrospective methodology used to collect clinical data (symptoms severity, previous GABHS infections) regarding the year before patients were included in the study [87,88]. More important, many patients had neuropsychiatric comorbidities (as in all PANDAS studies) and the study design failed to consider concomitant pharmacological treatments as possible sources of confounding. Current knowledge therefore seems insufficient to support routine antibiotic prophylaxis for the symptoms of PANDAS.

## Conclusion

Despite the encouraging results from recent studies that tested a possible autoimmune pathogenesis of PANDAS also at an intracellular level, and found in CaM kinase II a possible mediator of neuropsychiatric symptoms in this subset of OCD or TD patients, the validity of this nosologic entity is still questioned. The presence of anti-brain antibodies in a subset of patients with OCD, the promising results from immunomodulatory treatment in PANDAS and the possible association between some upper respiratory infections and the sudden onset of OCD, suggest a supportive role for immune triggers in some OCD subtypes. A research area that deserves further investigation regards the possible differences in the psychopathological characteristics of autoimmune-induced and non-autoimmune-induced OCD. Our findings in this review apart from confirming PANDAS as a distinct clinical entity, suggest that PANDAS is a useful research field that could open new insights into the pathogenesis of OCD, even in adults.

## Abbreviations

BBB: blood-brain barrier BBB; CaM kinase II: calcium-calmodulin dependent protein kinase II; CY-BOCS: children Yale-Brown obsessive compulsive scale; GABHS: group A β-hemolytic streptococcus; IVIG: intravenous immunoglobulin; OCD: obsessive-compulsive disorder; PANDAS: pediatric autoimmune neuropsychiatric disorders associated with streptococcal infections; TD: tic disorder

## Competing interests

The authors declare that they have no competing interests.

## Authors' contributions

GM, MP, GM, LT and MB conceived the manuscript and drafted it. All authors read and approved the final manuscript.
